# Phase II Study of Nivolumab and Ipilimumab for Treatment of Metastatic/Recurrent Adenoid Cystic Carcinoma (ACC) of all Anatomic Sites of Origin and Other Malignant Salivary Gland Tumors

**DOI:** 10.1002/cam4.70724

**Published:** 2025-04-01

**Authors:** Young Kwang Chae, Richard Duan, Liam Il‐Young Chung, Youjin Oh, Borislav Alexiev, Sangwon Shin, Sukjun Kim, Irene Helenowski, Maria Matsangou, Victoria Villaflor, Devalingam Mahalingam

**Affiliations:** ^1^ Department of Hematology and Oncology Northwestern University Chicago Illinois USA; ^2^ Feinberg School of Medicine Northwestern University Chicago Illinois USA; ^3^ Department of Surgical Pathology Northwestern University Chicago Illinois USA; ^4^ Lunit Seoul Republic of Korea; ^5^ Astellas Pharmaceuticals Northbrook Illinois USA; ^6^ Department of Medical Oncology City of Hope Los Angeles California USA

## Abstract

**Objective:**

Dual checkpoint inhibitor therapy with nivolumab and ipilimumab has been FDA approved for a number of cancer sites. However, its role in the treatment of ACC and non‐ACC salivary gland carcinomas (non‐ACC SGC) is not well established.

**Methods and Analysis:**

We performed Simon's two‐stage prospective single‐institution Phase II clinical trial of nivolumab with ipilimumab. Two cohorts were analyzed: patients with metastatic/recurrent ACC and patients with non‐ACC SGC. The primary endpoint was median progression‐free survival (PFS); secondary endpoints were overall response rate (ORR), overall survival (OS), and toxicity.

**Results:**

Patient enrollment was prematurely terminated due to funding constraints. In total, 19 patients with ACC and 5 patients with non‐ACC SGC were enrolled. The patients with ACC had a median OS of 30.0 months (95% CI 15.3‐NR months), a median PFS of 8.3 months (95% CI 5.5–30.0 months), and a disease control rate (DCR) of 53% (10/19). The ORR in the ACC group was 5% (CR 0%, *n* = 0; confirmed PR 5%, *n* = 1), with one patient having continued stable disease at the time of trial conclusion. The patients with non‐ACC SGC had a median OS of 10.4 months (95% CI 6.21‐NR months), a median PFS of 6.21 months (95% CI 2.83‐NR months), and a DCR of 40% (2/5). The ORR in this cohort was 0%.

**Conclusion:**

In patients with recurrent or metastatic ACC and non‐ACC SGC, the combination of nivolumab with ipilimumab resulted in moderate disease control. Further studies are warranted to validate our findings.

Trial number: NCT03146650.

## Graphical abstract

In patients with recurrent or metastatic adenoid cystic carcinoma (ACC) combination nivolumab with ipilimumab resulted in moderate disease control. Patients with non‐ACC salivary gland carcinomas did not see responses to treatment.

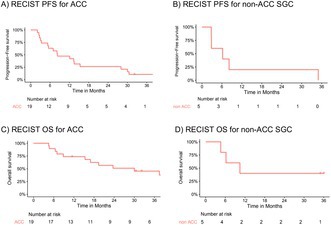

## 1 Introduction

Adenoid cystic carcinoma (ACC) is a subset of adenocarcinomas that primarily occur in the major and minor salivary glands. It affects 1200 individuals in the United States each year and comprises 10% of all cases of salivary gland tumors [1, 2]. The standard of treatment for localized ACC cases is surgical excision followed by radiotherapy [3]. However, despite treatment, ACC cases are characterized by perineural invasion and hematologic metastasis, most notably to the lungs [4]. This leads to frequent local and distant recurrences, resulting in a 10‐year survival rate of 61% [4]. For recurrent/metastatic ACC with > 5 metastases, current American Society for Clinical Oncology (ASCO) guidelines recommend treatment with multitargeted tyrosine kinase inhibitors (TKI) (e.g., lenvatinib and sorafenib) based on prior trials with modest rates of disease stabilization [5, 6, 7, 8, 9] (ASCO). For non‐ACC salivary gland cancers (non‐ACC SGC), targeted therapy based on molecular markers (e.g., AR, HER2, and NTRK) is indicated in the absence of suitable clinical trials.

Despite the persistence of ACC, it has an indolent course at the beginning. This makes it difficult to study clinical responses to chemotherapies, which have yet to demonstrate a positive effect on patient survival [1, 2]. Due to the ineffectiveness of cytotoxic chemotherapies [10], more research is needed for therapies that target different molecular pathologies of ACC. MYB translocations, c‐kit overexpression, EGFR overexpression, histone deacetylase aberrant activity, NOTCH1 mutations, and immunotherapy susceptibility are all examples, among other attributes [11].

Many experimental therapies thus far have undergone trials to attempt to exploit the mutations and biomarkers specific to ACC. The MYB transcription factor, the most well‐studied accessory of ACC oncogenesis, is mutated in about 70% of ACC patients [11], and a number of pharmaceutical targets along its signaling pathway have been identified. However, efforts to target this pathway have had little success, although a number of trials are still underway to further investigate this pathway as a therapeutic target [12]. An EGFR‐targeting drug, cetuximab, when combined with cisplatin, showed an overall response rate of 43% [10].

Vorinostat and chidamide, two histone deacetylase inhibitors, were shown to induce limited responses in ACC patients, even when the latter was used together with cisplatin [13, 14]. The NOTCH signaling pathway has been a target of a number of recent Phase I trials: a NOTCH1 antibody bronticuzumab, an upstream inhibitor of the NOTCH pathway CB‐103, and an inhibitor of NOTCH signaling AL101 [12]. However, these agents all showed minimal responses among patients in their respective trials, and research into targeted therapies remains crucial for improving the outcomes of ACC patients.

PD‐1 and CTLA‐4 are two cell surface receptors that serve as inhibitory checkpoints for activated T cells when bound. Two immune checkpoint inhibitors, nivolumab and ipilimumab, target PD‐1 and CTLA‐4 respectively to prevent abrogation of T cell activation [15]. Although a trial of nivolumab alone did not show much efficacy, with overall response rates below 10% [16], the nivolumab/ipilimumab combination held some promise in the treatment of ACC. This combination of immune checkpoint inhibitors has already been FDA‐approved for the treatment of metastatic or recurrent NSCLC, melanoma, renal cell carcinoma, hepatocellular carcinoma, and microsatellite instability‐high/mismatch repair deficient colorectal cancers [15]. Furthermore, past nivolumab/ipilimumab trials for adenoid cystic salivary and non‐ACC SGC have shown dramatic tumor regression changes in the patients who did exhibit positive responses [17, 18]. The objective of this study is to further elucidate the efficacy of the nivolumab/ipilimumab combination in the treatment of ACC and to elucidate differences in responses between immunogenic and nonimmunogenic cases of ACC.

## 2 Methods

### 2.1 Patients

Eligibility criteria include a confirmed diagnosis of metastatic/recurrent ACC from any anatomical origin or non‐ACC neoplasms of a salivary gland; noncandidacy for curative surgical or radiation therapy; measurable disease in accordance with RECIST v1.1; at least 18 years of age; an Eastern Cooperative Oncology Group status of 0–2 with 3 allowed if directly secondary to ACC; adequate bone marrow and organ function; no investigational or chemotherapies administered within 28 days or radiation within 7 days of study treatment; and no history of autoimmune disease that may affect organ function or require immunosuppressive treatment. Progressive ACC was not part of the inclusion criteria because our enrolled patients were already refractory to standard‐of‐care therapeutic options and had an unmet need for treatment. Study approval was given by the institutional IRB in accordance with federally mandated regulations. Patient informed consent was individually obtained in written form, and patients were not involved in the design and implementation of this study.

### 2.2 Intervention

Patients were treated with nivolumab at 240 mg intravenously every 2 weeks for 16 weeks, then starting on Cycle 2 Day 29, given at 480 mg IV every 4 weeks. Ipilimumab was administered at 1 mg/kg intravenously every 6 weeks. Patients were assessed for response every 12 weeks (+/− 7 days). Treatment was provided until disease progression, intercurrent illnesses preventing further treatment, treatment delays greater than 42 days, patient withdrawal, or unacceptable toxicities and adverse events. All patients who received at least one dose of nivolumab and had a radiologic evaluation of disease were evaluated for efficacy endpoints. Adverse events were graded using version 4.0 of the National Cancer Institute's Common Terminology Criteria for Adverse Events and followed up to 30 days after treatment cessation or initiation of a new treatment.

### 2.3 Endpoints

A Simon two‐stage optimum Phase II design was used for this trial with main outcomes of PFS at 6 and 12 months (supplemental methods). The trial was initially designed and powered for patients with ACC, with an exploratory group of patients with non‐ACC SGC. In the first stage, 15 patients with ACC were enrolled, with accrual to the next stage contingent upon fewer than 7 of the 15 patients withdrawing from the study for reasons unrelated to withdrawal of consent within a timespan of 24 weeks after drug administration. In the actual trial, 10/15 patients remained after 24 weeks. Thus, the trial entered the second stage, where 43 patients were to be recruited. Due to funding constraints, trial enrollment concluded after 9 additional patients joined.

Patients with recurrent/metastatic non‐ACC SGC were included as an exploratory group with separate statistical analysis and safety analysis. The initial plan for the exploratory group was for 10 patients to initially be enrolled, with the accrual of an additional 10 patients contingent on 2 or fewer of the initial 10 non‐ACC patients withdrawing after 24 weeks of treatment. However, only 5 patients with recurrent/metastatic non‐ACC SGC were ultimately enrolled due to funding constraints.

The primary endpoint of the study was median progression‐free survival (PFS) and PFS at 6 months based on blinded central radiology review every 12 weeks using RECIST and immune‐related RECIST (irRECIST) criteria version 1.1 in parallel (PFS‐6 and iPFS‐6, respectively). Secondary endpoints include assessment of the overall response rate (ORR), clinical benefit rate (CBR), overall survival (OS), and progression‐free survival (PFS) using RECIST and irRECIST criteria.

PFS was measured from the date of treatment initiation to disease progression or death from any cause, whichever came first. Patients without documented disease progression on their last follow‐up were censored at the date of last contact. OS was measured from the date of treatment initiation to the date of death from any cause, with patients last known to be alive censored at the date of last contact. PFS and OS were estimated using the Kaplan–Meier method and compared using log‐rank tests. All analyses were done using R version 4.0.3.

### 2.4 PD‐L1 Immunohistochemistry

PD‐L1 expression was assessed using the Ventana PD‐L1 (SP263) Assay with the OptiView DAB IHC Detection Kit (OptiView). Formalin‐fixed paraffin‐embedded tissue slides were stained with the above kits and viewed under light microscopy, with percent staining visually quantified by a pathologist blinded to the study results.

### 2.5 Next‐Generation Sequencing

Next‐generation sequencing was performed using the Tempus xT 596 gene panel. Patient tumor samples were sequenced with matched blood samples as controls. Tumor mutational burden (TMB), single nucleotide variants, insertions or deletions, copy number variants, and translocations in 21 genes were all measured using DNA sequencing. A total of 43 DNA sites were analyzed for microsatellite instability status. Sequencing was performed to 500x depth for tumor samples and 150x for normal samples.

### 2.6 Tumor‐Infiltrating Lymphocyte Analysis

Whole H&E‐stained slides were scanned and analyzed by Lunit SCOPE IO, an artificial intelligence model that is trained to analyze the distribution of tumor‐infiltrating lymphocytes (TILs) [19]. Whole slide images were divided into 1‐mm^2^‐sized grids and given immune phenotype classifiers based on the concentration of TILs within each grid. Furthermore, the proportion of each grid‐level classifier to the total number of analyzed grids in the slide was analyzed to determine an overall inflamed score.

## 3 Results

### 3.1 Patient Characteristics

Due to premature trial termination resulting from funding constraints, a total of 24 patients were included: 19 patients in the ACC cohort and 5 patients in the other salivary neoplasm cohort. Histologic subtypes among the non‐ACC SGC group included adenocarcinoma NOS (*N* = 2), adenocarcinoma with clear cell features (*N* = 1), mucoepidermoid carcinoma (*N* = 1), and salivary duct carcinoma (*N* = 1). Among the ACC patients (*n* = 19), the median age was 56.0 years, with 47.4% of patients being men. For the non‐ACC SGC cohort (*n* = 5), the median age was 62.0 years, with 60.0% of patients being men (Table 1). Complete blood counts prior to treatment initiation were collected for 23 patients, and molecular characterization performed as part of routine care was available for four patients.

**TABLE 1:** Summary of patient demographics and characteristics.

	Overall, *N* = 24	Non‐ACC, *N* = 5	ACC, *N* = 19
Mean age (SD)	56.3 (14.7)	60.6 (12.4)	55.2 (15.3)
Male, *n* (%)	12 (50.0%)	3 (60.0%)	9 (47.4%)
BMI (SD)	25.4 (3.8)	27.8 (4.3)	24.8 (3.5)
ECOG status			
0	16 (66.7%)	4 (80.0%)	12 (63.2%)
1	8 (33.33%)	1 (20.0%)	7 (36.8%)
Location			
Large salivary gland	12 (50.0%)	4 (80.0%)	8 (42.1%)
Sinus	2 (8.3%)	0 (0.0%)	2 (10.5%)
Lacrimal gland	3 (12.5%)	0 (0.0%)	3 (15.8%)
Base of tongue	2 (8.3%)	0 (0.0%)	2 (10.5%)
Retromolar trigone	1 (4.2%)	1 (4.2%)	0 (0.0%)
Nasal cavity	1 (4.2%)	0 (0.0%)	1 (5.3%)
Mandible	1 (4.2%)	0 (0.0%)	1 (5.3%)
Ear	1 (4.2%)	0 (0.0%)	1 (5.3%)
Bartholin gland	1 (4.2%)	0 (0.0%)	1 (5.3%)
Histology of non‐ACC SGC			
Adenocarcinoma NOS		2 (40.0%)	
Adenocarcinoma w/clear cell features		1 (20.0%)	
Mucoepidermoid carcinoma		1 (20.0%)	
Salivary duct carcinoma		1 (20.0%)	
Staging			
Recurrent	1 (4.2%)	0 (0.0%)	1 (5.3%)
IV	23 (95.8%)	5100.0%)	18 (94.7%)
Prior therapies			
Surgical resection	20 (83.3%)	5 (100.0%)	15 (78.9%)
Radiation	17 (70.8%)	3 (60.0%)	14 (73.7%)
Concurrent chemoradiation	7 (29.2%)	2 (40.0%)	5 (26.3%)
Chemotherapy	6 (25.0%)	2 (40.0%)	4 (21.1%)
TKI	4 (16.7%)	0 (0.0%)	4 (21.1%)
mTOR inhibitor	2 (8.3%)	1 (20.0%)	1 (5.3%)

Abbreviations: *N*, number; non‐ACC SGC, nonadenoid cystic carcinoma salivary gland carcinoma; SD, standard deviation; TKI, tyrosine kinase inhibitor.

### 3.2 Outcomes

**FIGURE 1:**
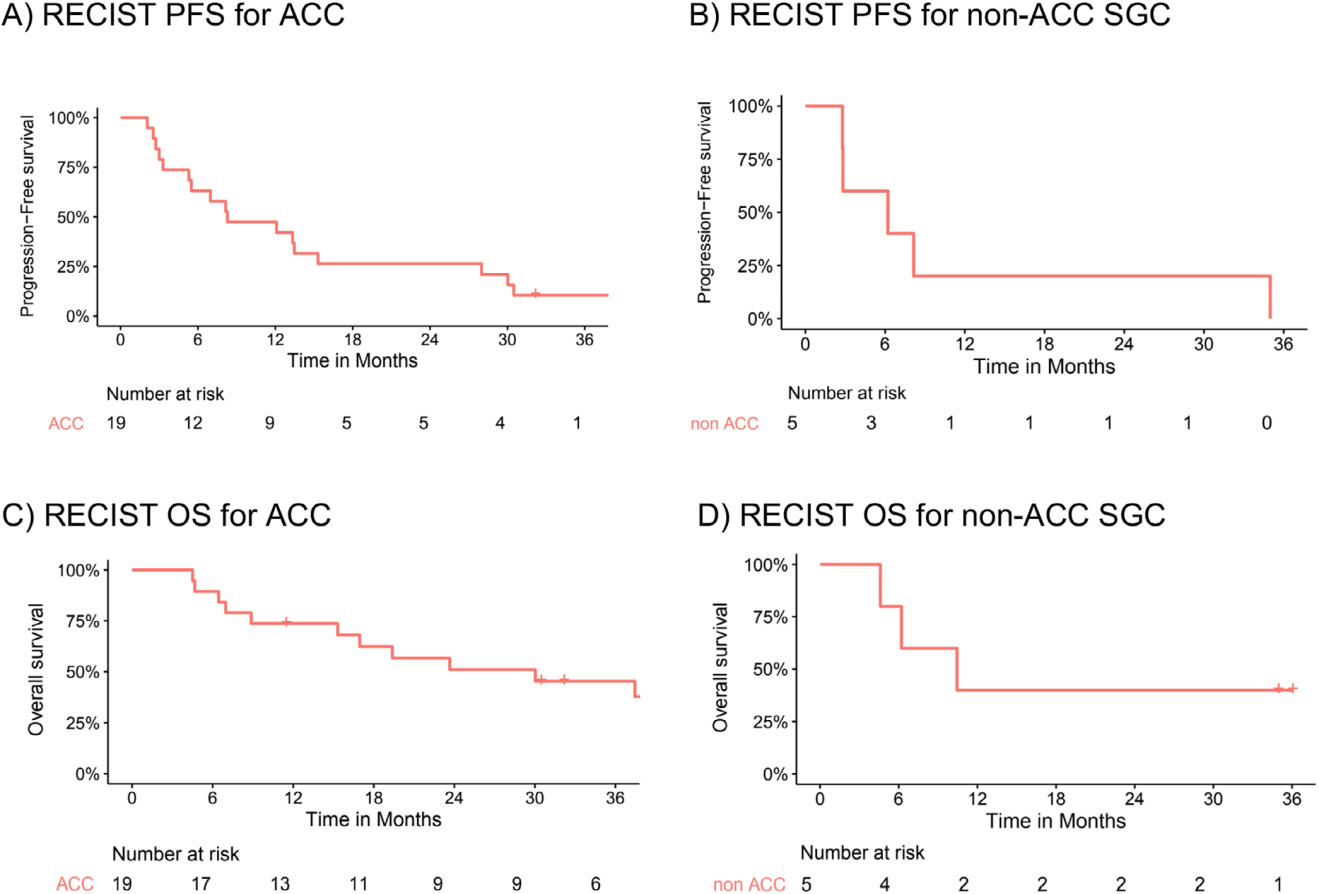
Progression‐free survival (PFS) and overall survival (OS) per RECIST criteria displayed on Kaplan–Meier curves.

**FIGURE 2:**
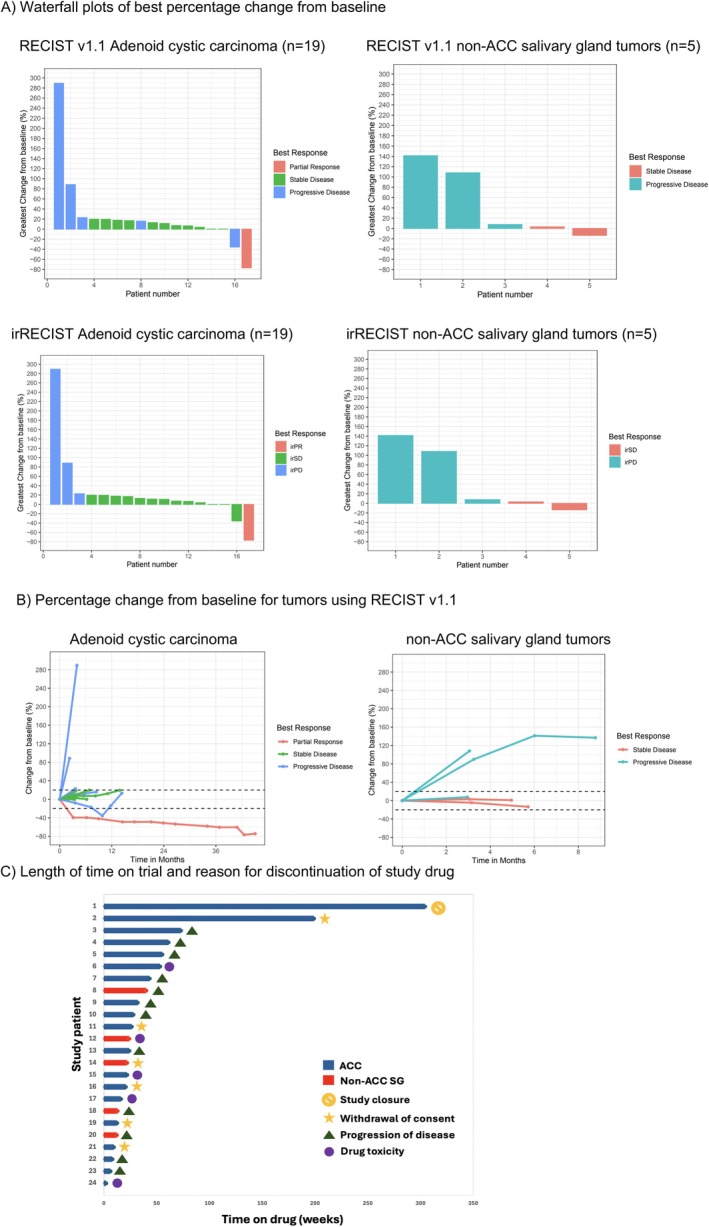
(A) Waterfall plots of best percentage change from baseline. (B) Percentage change from baseline for tumors using RECIST v1.1. Outcomes of patients with metastatic/recurrent ACC or non‐ACC salivary gland cancers treated with nivolumab and ipilimumab. (A) OS and PFS displayed on Kaplan–Meier curve. (B) Waterfall plot depicting RECIST or irRECIST progression, stable disease, or partial response. (C) Spider plot displaying percentage change in longest tumor diameters over time. (C) Length of time on trial and reason for discontinuation of study drug. (A) Waterfall plot depicting RECIST or irRECIST progression, stable disease, or partial response. (B) Spider plot displaying percentage change in longest tumor diameters over time. (C) Swimmer plot detailing length of time each patient spent on the trial drug and their respective reason for discontinuation.

Among the 19 ACC patients, the median OS was 30.0 months (95% confidence interval (CI) 15.3 months to not reached), the median PFS was 8.3 months (95% CI 5.5–30.0 months), and the disease control rate (DCR) was 42.1% (8/19) using RECIST measurements (Figure 1, Table 2). The PFS‐6 and iPFS‐6 were both 63% (12/19), whereas OS at 6 months was 89% (17/19). The overall response rate (ORR) in the ACC group was 5% (CR 0%, *n* = 0; confirmed PR 5%, *n* = 1) using both RECIST and irRECIST criteria. Responses of note include a durable PR lasting over 48 months and a patient with ongoing SD lasting over 59 months (Figure 2).

**TABLE 2:** Best responses.

RECIST responses			
Characteristic	Overall, *N* = 24	Non‐ACC, *N* = 5	ACC, *N* = 19
CR	0 (0.00%)	0 (0.00%)	0 (0.00%)
PR	1 (4.17%)	0 (0.00%)	1 (5.26%)
SD	13 (54.17%)	2 (40.00%)	11 (57.89%)
PD	9 (37.50%)	3 (60.00%)	6 (31.58%)
N/A	1 (4.17%)	0 (0.00%)	1 (5.26%)
DCR*	10 (41.7%)	2 (40.0%)	8 (42.1%)

irRECIST responses			
CR	0 (0.00%)	0 (0.00%)	0 (0.00%)
PR	1 (4.17%)	0 (0.00%)	1 (5.26%)
SD	15 (62.5%)	2 (40.00%)	13 (68.4%)
PD	6 (25.0%)	3 (60.00%)	3 (15.8%)
N/A	2 (8.33%)	0 (0.00%)	2 (10.5%)
DCR*	11 (45.8%)	2 (40.0%)	9 (47.3%)

*Note:* Disease control rate: CR + PR + SD lasting over 24 weeks.

Abbreviations: CR, complete response; PD, progressive disease; PR, partial response; SD, stable disease.

For the five patients with non‐ACC SGC, the median OS was 10.4 months (95% CI 6.21 months to not reached), the median PFS time was 6.21 months (95% CI 2.83 months to not reached), and the DCR was 40% (2/5). PFS‐6 and iPFS‐6 were both 40% (2/5) with an OS at 6 months of 80% (4/5) and ORR of 0% using both RECIST and irRECIST criteria (Figure 1, Table 2).

### 3.3 Toxicities

All 24 of our patients experienced a treatment‐related adverse event (AE), the most common of which include fatigue (54%, *n* = 13), lymphocytopenia (46%, *n* = 11), and anemia (42%, *n* = 10). 45.8% of patients (*n* = 11) experienced a Grade III–IV treatment‐related AE; none of the patients had Grade V treatment‐related AE (Table 3, full list of all AEs in Table S1). A couple of serious treatment‐related adverse events were noted, including adrenal insufficiency (*n* = 2, one Grade II and one Grade II), ALT elevation (*n* = 1, Grade IV), AST elevation (*n* = 1, Grade IV), anorexia (*n* = 1, Grade III), and colitis (*n* = 1, Grade III). Fortunately, no patient deaths were associated with the study. Lastly, out of all patients who experienced an AE, 12.5% (*n* = 3) led to discontinuation of treatment.

**TABLE 3:** Immune‐related adverse events (AEs) separated by grades (I/II vs. III/IV/V).

	All Grades	Grade III/IV
Any	24 (100%)	11 (45.83%)
Led to discontinuation	3 (12.5%)	3 (12.5%)
Led to death	0 (0.0%)	0 (0.0%)
Immune‐Related AEs		
Fatigue	13 (54.17%)	1 (4.17%)
Lymphocyte count decreased	11 (45.83%)	4 (16.67%)
Anemia	10 (41.67%)	2 (8.33%)
Aspartate aminotransferase increased	9 (37.5%)	1 (4.17%)
Nausea	8 (33.33%)	0 (0%)
Diarrhea	6 (25%)	1 (4.17%)
Hypothyroidism	6 (25%)	0 (0%)
Anorexia	5 (20.83%)	1 (4.17%)
Cough	5 (20.83%)	0 (0%)
Alanine aminotransferase increased	4 (16.67%)	1 (4.17%)
Pruritus	4 (16.67%)	0 (0%)
Weight loss	4 (16.67%)	1 (4.17%)
Adrenal insufficiency	3 (12.5%)	1 (4.17%)
Dyspnea	3 (12.5%)	0 (0%)
Hyperglycemia	3 (12.5%)	0 (0%)
Rash maculo‐papular	3 (12.5%)	1 (4.17%)
Abdominal pain	2 (8.33%)	0 (0%)
Alkaline phosphatase increased	2 (8.33%)	1 (4.17%)
Colitis	2 (8.33%)	1 (4.17%)
Creatinine increased	2 (8.33%)	0 (0%)
Headache	2 (8.33%)	0 (0%)
Hyperthyroidism	2 (8.33%)	0 (0%)
Hyponatremia	2 (8.33%)	0 (0%)
Mucositis oral	2 (8.33%)	1 (4.17%)
Myalgia	2 (8.33%)	0 (0%)
Nasal congestion	2 (8.33%)	0 (0%)
Productive cough	2 (8.33%)	0 (0%)
Vomiting	2 (8.33%)	0 (0%)
Alopecia	1 (4.17%)	0 (0%)
Arthralgia	1 (4.17%)	0 (0%)
Chills	1 (4.17%)	0 (0%)
Constipation	1 (4.17%)	0 (0%)
Dysphagia	1 (4.17%)	0 (0%)
Edema face	1 (4.17%)	0 (0%)
Edema limbs	1 (4.17%)	0 (0%)
Enterocolitis	1 (4.17%)	0 (0%)
Flu‐like symptoms	1 (4.17%)	0 (0%)
Flushing	1 (4.17%)	0 (0%)
Gastroesophageal reflux disease	1 (4.17%)	0 (0%)
Hypercalcemia	1 (4.17%)	0 (0%)
Hyperhidrosis	1 (4.17%)	0 (0%)
Hyperkalemia	1 (4.17%)	0 (0%)
Hypertension	1 (4.17%)	0 (0%)
Hypoalbuminemia	1 (4.17%)	0 (0%)
Hypokalemia	1 (4.17%)	1 (4.17%)
Hypophosphatemia	1 (4.17%)	1 (4.17%)
Hypotension	1 (4.17%)	0 (0%)
Infusion‐related reaction	1 (4.17%)	0 (0%)
Joint range of motion decreased	1 (4.17%)	0 (0%)
Lipase increased	1 (4.17%)	0 (0%)
Lymphocyte count increased	1 (4.17%)	0 (0%)
Muscle weakness lower limb	1 (4.17%)	0 (0%)
Noncardiac chest pain	1 (4.17%)	0 (0%)
Pain in extremity	1 (4.17%)	0 (0%)
Platelet count decreased	1 (4.17%)	0 (0%)
Presyncope	1 (4.17%)	0 (0%)
Serum amylase increased	1 (4.17%)	1 (4.17%)
Sinus tachycardia	1 (4.17%)	0 (0%)

### 3.4 Cellular and Molecular Characterization

Prior to treatment initiation, 23 of the 24 patients had complete blood counts drawn, from which medians of the platelet count (246 K/uL), neutrophil count (4.3 K/uL), lymphocyte count (1.2 K/uL), and neutrophil‐to‐lymphocyte ratio (3.36) were calculated. Across both ACC and salivary neoplasm cohorts, platelet counts above the median were significantly associated with better OS (*p* = 0.032) and PFS (*p* = 0.046) (Figure S1). Additionally, although not significant, there was a slight trend for improved OS and PFS among patients with neutrophil‐to‐lymphocyte ratios greater than five (OS *p* = 0.42, PFS *p* = 0.25) and above‐median neutrophil counts (OS *p* = 0.24, PFS *p* = 0.18).

Four of the 24 patients additionally underwent next‐generation sequencing (NGS), all of whom had at least two pathological aberrations detected. Of special note, two MYB‐NFIB translocations, one NOTCH1 Cys1383 frameshift mutation, one MTOR N1760K mutation, one FGFR2 overexpression, one HRAS overexpression, and one PDGFRA copy number gain were detected (Table S2). None of the patients had high levels of tumor mutational burden detected. Additionally, the two patients who also had available PDL1 immunohistochemistry had < 1% staining.

Six of the 24 patients had H&E‐stained whole slide images (WSIs) available for analysis using an artificial intelligence (AI)‐powered WSI analyzer, Lunit SCOPE IO (Lunit, Seoul, Republic of Korea), which analyzes the spatial distribution of tumor‐infiltrating lymphocytes (TILs) throughout the WSI to predict responsiveness to ICI [19, 20]. Among these 6 patients, the three who reached SD had the lowest inflamed scores and were categorized as immune‐desert, corresponding to below‐threshold concentrations of TILs throughout the tumor microenvironment (Table 4). In contrast, the remaining three patients, who had PD as the best response, showed diverse phenotypes: two were categorized as immune‐excluded, representing higher TIL concentration in the cancer stroma area, and one case was classified as immune‐desert, despite having relatively higher TIL concentration in both the cancer and cancer stroma areas.

**TABLE 4:** Immune phenotype of patients with available H&E slides.

Patient #	Immune phenotype^a^	IS^b^	IES^b^	IDS^b^	PD‐L1 expression	ACC status	Best response
5	Immune‐desert	18.29	28.05	56.66	#N/A	ACC	PD
9	Immune‐desert	1.08	4.69	94.22	#N/A	ACC	SD
11	Immune‐desert	0	0.81	99.19	#N/A	ACC	SD
23	Immune‐desert	0.88	29.23	69.89	15%	non ACC	SD
22	Immune‐excluded	25.36	33.93	40.71	#N/A	non ACC	PD
25	Immune‐excluded	1.13	54.80	44.07	< 1%	non ACC	PD

Abbreviations: ACC, adenoid cystic carcinoma; AI, artificial intelligence; IDS, immune‐desert score; IES, immune‐excluded score; IP, immune phenotype; IS, inflamed score; N/A, not available; PD, progressive disease; PD‐L1, programmed cell death ligand 1; SD, stable disease; TIL, tumor‐infiltrating lymphocytes; WSI, whole‐slide image.

^a^ If the IS is ≥ 33.3%, the IPs at the WSI level were determined to be “Inflamed.” Similarly, if the IES is ≥ 33.3%, the WSI‐level IPs were determined to be “Immune‐excluded,” while all remaining samples were categorized as “Immune‐desert.”

^b^ Immune phenotypes (IPs) and inflamed scores were defined using an AI‐powered WSI analyzer, Lunit SCOPE IO [19, 20]. Analysis was performed on 1‐mm^2^‐sized grids derived from WSI, classifying the IPs of each grid as follows: Immune‐desert indicates below‐threshold levels of tumor‐infiltrating lymphocytes (TILs) in both the cancer area and cancer stroma, immune‐excluded indicates above‐threshold levels of TILs in the cancer stroma and below‐threshold levels in the cancer area, and inflamed indicates above‐threshold TIL levels in the cancer area. The inflamed score (IS), immune‐excluded score (IES), and immune‐desert score (IDS) were calculated as the proportion of each grid‐level phenotype to the total analyzed grids in WSI.

## 4 Discussion

In this study, nivolumab and ipilimumab had a moderate efficacy for patients with recurrent/metastatic ACC, while patients with non‐ACC SGC did not see responses to the combined checkpoint inhibition. This trial's results complement a few previous studies that evaluated nivolumab/ipilimumab as treatment for patients with recurrent/metastatic ACC and/or non‐ACC SGC. Compared to the 5% (1/19) ORR in our study's ACC cohort, our previous study and another prior study found ORRs of 4% (1/26) and 6% (2/32), respectively, with comparably durable responses (median PFS 4.4 months vs. 8.3 months in this study) in ACC patients [18, 21].

In our study's non‐ACC salivary carcinoma cohort, we saw a 0% (0/5) ORR, whereas prior trials saw 16% (5/32) and 9% (3/35) ORRs in patients with recurrent/metastatic non‐ACC SGC also with comparably durable responses (median PFS 2.2 months vs. 6.21 months in our study) [18, 21]. The few durable responses seen in our current and prior trials were mirrored in a case report in which a patient with recurrent and metastatic parotid gland cystadenocarcinoma had a complete response to nivolumab and ipilimumab [22]. Although our limited non‐ACC SGC cohort's outcomes fall in line with previous studies, our small cohort size should not allow for conclusions to be made.

These infrequent cases of exceptional responses highlight the need for understanding the reasons underlying the remarkable responses. One of the previous ACC trials found that exceptional responders had greater pretreatment intratumoral T‐cell clonal expansion, likely related to the greater immune checkpoint response [21]. Another prior study suggests the existence of two molecular subgroups of ACC (ACC‐I and ACC‐II) with dramatically differing prognoses that are driven by mutated signaling pathways involving MYC hyperactivation versus p63 suppression [23]. Although our patients did not undergo molecular analyses to support these findings, the one patient with ACC who displayed a positive response did have NGS‐based testing done. Their testing identified two frameshift mutations in the *BCOR* gene. BCOR is associated with improved immune checkpoint inhibitor responses when mutated in different types of lung cancer, and therefore could partly explain the exceptional benefit the patient saw with nivolumab/ipilimumab treatment [24].

Prior studies have also evaluated PD‐1/PD‐L1 inhibitor monotherapy for patients with both ACC and non‐ACC SGC. Among the ACC‐related studies, a trial comparing pembrolizumab with and without radiation for patients with recurrent/metastatic ACC saw an ORR of 0% for all patients included [25]. A separate trial evaluating nivolumab included 46 patients with recurrent/metastatic ACC and saw an 8.7% ORR [16]. These studies yielded comparable ORRs to our study and other prior dual checkpoint inhibitor trials for recurrent/metastatic ACC. However, their median PFS (4.9 and 6.6 for pembrolizumab only) was lower than that seen in our study (8.3 months), suggesting a possible role of CTLA‐4 inhibitors like ipilimumab in extending the responses that patients have to immunotherapy [26, 27].

Alongside the checkpoint inhibitor monotherapy studies, recent trials evaluated TKI therapy for recurrent/metastatic ACC with and without immune checkpoint inhibition. The first study evaluated axitinib, a VEGFR inhibitor, as monotherapy for patients with ACC. They saw an ORR of 0% among 54 patients, but the primary endpoint of 6‐month PFS rate was significantly improved at 73% in the axitinib group and 23% in the observation group [5]. A following study investigated axitinib with avelumab, a PD‐L1 inhibitor. Among 28 patients, they achieved an ORR of 18% with a median PFS of 7.3 months, reaching their primary ORR endpoint [28]. A separate trial investigated lenvatinib, a multitargeted TKI, alongside pembrolizumab. This trial achieved an ORR of 6% out of 17 patients, failing to meet criteria for progression to the second stage of accrual [29].

Non‐ACC SGC were also investigated in past trials, including nivolumab monotherapy for patients with recurrent or metastatic non‐ACC SGC that resulted in ORRs ranging from 4.2% to 8.7% [16, 30, 31]. Another subset of trials evaluated pembrolizumab monotherapy, achieving ORRs of 12% and 4.6% in patients with recurrent/metastatic non‐ACC SGC [32, 33]. Although PD‐1/PD‐L1 inhibitor monotherapy yielded comparable ORRs to our study, their reported median PFS was also lower (1.6–4.9 months) compared to our study (6.1 months) [16, 30, 31, 32, 33]. This again suggests a role of ipilimumab in prolonging responses to PD‐1/PD‐L1 therapy, although durable positive responses over 48 weeks were seen in some monotherapy studies [22, 27, 33].

In addition to data on overall responses, we explored pretreatment cell counts for platelets, neutrophils, and lymphocytes for their correlations with immunotherapy treatment outcomes. Platelet counts especially were notable for their association with treatment outcomes: patients with below‐median platelet counts had a significant association with increased PFS and OS (*p* = 0.032 and 0.046 respectively) with a hazard ratio of 3.60 (95% CI 1.11–11.7) for increased OS. The protective association with low platelets has been previously described, as platelets have been known to promote tumor progression through angiogenesis and adhesion molecule production [34]. Furthermore, the association between elevated platelet counts and neutrophil/lymphocyte ratios with poor outcomes has already been described for many other cancers [35]. However, their replicability in our study tells us that the host immune milieu is important no matter the tumor type, including for ACC and non‐ACC SGC.

We also explored a subset of our patients with available tissue slides for TIL distribution and found that all three patients with lower TIL concentrations reached SD, whereas the other three patients only had PD while on ICI treatment. Previous literature describes how immune checkpoint blockade is correlated with an increase in intratumoral lymphocyte concentration [36]. In the case of CTLA‐4 inhibition, pretreatment tumor T‐lymphocyte levels were not found to correlate with outcomes in patients with melanoma [28]. Instead, clinical activity was correlated with the increase in T lymphocyte levels after treatment. In our study, patients only had pretreatment samples analyzed. Thus, a possible explanation for patients having more durable responses with lower pre‐ICI treatment lymphocyte levels is that they benefited from T lymphocyte recruitment after combined PD‐1/CTLA‐4 inhibition. Nonetheless, there is a need for further investigation using a larger cohort size.

Our study is an innovative basket trial whose main strengths include the NGS and histopathologic data available for our unique positive responder and a handful of other patients. This lends a better understanding of the few patients who do respond to immune checkpoint therapy. One main limitation in working with patients who have ACC and non‐ACC SGC is that their cancer biology is heterogeneous and contains many distinct histologic subtypes that are not controlled for, making it difficult to draw similarities between studies. Another weakness is the lack of a control group. Due to the naturally indolent nature of ACC growth and the tendency for delayed recurrence after treatment, a control group would help put this trial's patient responses into perspective of the specific clinical courses commonly seen in ACC patients [11]. Similarly, disease control rate may not be the ideal endpoint for an ACC trial given the slow growth pattern. Additional limitations include small sample sizes, limited molecular characterization of patient samples, and the lack of patient randomization.

In conclusion, ACC and non‐ACC SGC compose a group of rare heterogeneous malignancies that have no currently FDA‐approved treatment options. Treatment with nivolumab and ipilimumab results in modest response rates for patients with ACC, but not non‐ACC SGC, with results comparable to previously conducted studies on dual checkpoint inhibition [17, 37] and PD‐1/PD‐L1 monotherapy [16, 25, 30, 31, 32, 33]. We investigated further into a subset of our patients to examine their genomic, transcriptomic, and hematologic profiles. The one patient who had a PR was found to express two mutations that have established associations with the immune microenvironment, suggesting a possible cause for their elevated response. Further studies are warranted to investigate ACC and non‐ACC SGC biology and the factors that predict immune checkpoint therapy outcomes.

## Author Contributions

**Young Kwang Chae:** conceptualization (lead), data curation (equal), funding acquisition (lead), investigation (lead), methodology (equal), project administration (lead), writing – original draft (equal), writing – review and editing (equal). **Richard Duan:** data curation (equal), formal analysis (equal), software (equal), writing – original draft (lead), writing – review and editing (lead). **Liam Il‐Young Chung:** formal analysis (equal), project administration (equal), writing – original draft (equal), writing – review and editing (equal). **Youjin Oh:** data curation (equal), formal analysis (equal), software (equal), writing – original draft (equal), writing – review and editing (equal). **Borislav Alexiev:** data curation (equal), formal analysis (equal). **Sangwon Shin:** formal analysis (equal), software (equal), writing – review and editing (equal). **Sukjun Kim:** formal analysis (equal), software (equal), writing – review and editing (equal). **Irene Helenowski:** data curation (equal), formal analysis (equal), software (equal), writing – review and editing (equal). **Maria Matsangou:** conceptualization (equal), investigation (equal), project administration (equal), writing – review and editing (equal). **Victoria Villaflor:** conceptualization (equal), data curation (equal), investigation (equal), methodology (equal), project administration (equal), writing – review and editing (equal). **Devalingam Mahalingam:** conceptualization (equal), investigation (equal), methodology (equal), project administration (equal), writing – review and editing (equal).

## Ethics Statement

Informed consent was obtained directly from patients for participation in this study and its publication. Study approval was given by the Northwestern University Institutional Review Board Office in accordance with federally mandated regulations. Patient informed consent was individually obtained in written form, and patients were not involved in the design, conduct, reporting, or dissemination of this study.

## Conflicts of Interest

Funding, nivolumab, and ipilimumab were provided by Bristol Myers Squibb. YKC reports grants from AbbVie, BMS, Biodesix, Freenome, Predicine, and Roche/Genentech, as well as personal fees from Neogenomics, AstraZeneca, Foundation Medicine, Guardant Health, Merck, Boehringer Ingelheim, Biodesix, ImmuneOncia, Lilly Oncology, Takeda, Lunit, Jazz Pharmaceuticals, NeoImmunTech, Tempus, BMS, Regeneron, and Eisai outside the submitted work. VF reports stock and other ownership interests at Johnson & Johnson/Janssen, consulting or advisory roles at ARIAD/Takeda, AstraZeneca, Genentech/Roche, Bristol Myers Squibb, and Novocure, research funding from Takeda Science Foundation, and travel, accommodations, and expenses from ARIAD/Takeda, AstraZeneca, Bristol Myers Squibb, and Novocure. DM has received research funding from Amgen, Merck, Oncolytics, and Rafael; scientific advisory board for Actuate, Qurient, OncoOne, and an advisory/speaker bureau for Amgen, BMS, Eisai, and Exelixis; has received funding paid to their institution from Acepodia, Actuate Therapeutics, ADC Therapeutics, Amgen, AVEO, Bayer, Blueprint Medicines, BMS, BioNTech, Dialectic Therapeutics, Epizyme, Fujifilm, ImmuneSensor, Immune‐Onc Therapeutics, Leap Therapeutics, Lycera Corp, Merck, Millennium, MiNA Alpha, NGM Biopharmaceuticals, Novartis, Oncolytics, Orano Med, Puma, Qurient, Rafael, Repare Therapeutics, Triumvira Immunologics, Vigeo Therapeutics, and Warewolf Therapeutics. SS and SK are employees of Lunit. MM is an employee of Astellas Pharmaceuticals. RD, LIC, YO, BA, and IH report no conflicts of interest.

## Supporting information




Figure S1.

Table S1.

Table S2.

Data S1.

## Data Availability

All data pertinent to our study has been included in this manuscript or uploaded alongside it as Supporting Information.

## References

[1] A. Coca‐Pelaz , J. P. Rodrigo , P. J. Bradley , et al., “Adenoid Cystic Carcinoma of the Head and Neck‐‐An Update,” Oral Oncology 51 (2015): 652–661, 10.1016/j.oraloncology.2015.04.005. 25943783

[2] P. M. Dillon , G. R. Petroni , B. J. Horton , et al., “A Phase II Study of Dovitinib in Patients With Recurrent or Metastatic Adenoid Cystic Carcinoma,” Clinical Cancer Research 23 (2017): 4138–4145, 10.1158/1078-0432.CCR-16-2942. 28377480 PMC5540767

[3] Y. Chen , Z.‐Q. Zheng , F.‐P. Chen , et al., “Role of Postoperative Radiotherapy in Nonmetastatic Head and Neck Adenoid Cystic Carcinoma,” Journal of the National Comprehensive Cancer Network 18 (2020): 1476–1484, 10.6004/jnccn.2020.7593. 33152705

[4] S. Jang , P. N. Patel , R. J. Kimple , and T. McCulloch , “Clinical Outcomes and Prognostic Factors of Adenoid Cystic Carcinoma of the Head and Neck,” Anticancer Research 37 (2017): 3045–3052, 10.21873/anticanres.11659. 28551643 PMC7238770

[5] E. J. Kang , M.‐J. Ahn , C.‐Y. Ock , et al., “Randomized Phase II Study of Axitinib Versus Observation in Patients With Recurred or Metastatic Adenoid Cystic Carcinoma,” Clinical Cancer Research 27 (2021): 5272–5279, 10.1158/1078-0432.CCR-21-1061. 34315722

[6] D. J. Thomson , P. Silva , K. Denton , et al., “Phase II Trial of Sorafenib in Advanced Salivary Adenoid Cystic Carcinoma of the Head and Neck,” Head & Neck 37 (2015): 182–187, 10.1002/hed.23577. 24346857

[7] L. D. Locati , F. Perrone , B. Cortelazzi , et al., “A Phase II Study of Sorafenib in Recurrent and/or Metastatic Salivary Gland Carcinomas: Translational Analyses and Clinical Impact,” European Journal of Cancer 69 (2016): 158–165, 10.1016/j.ejca.2016.09.022. 27821319

[8] V. Tchekmedyian , E. J. Sherman , L. Dunn , et al., “Phase II Study of Lenvatinib in Patients With Progressive, Recurrent or Metastatic Adenoid Cystic Carcinoma,” Journal of Clinical Oncology 37 (2019): 1529–1537, 10.1200/JCO.18.01859. 30939095 PMC6599407

[9] J. L. Geiger , N. Ismaila , B. Beadle , et al., “Management of Salivary Gland Malignancy: ASCO Guideline,” Journal of Clinical Oncology 39 (2021): 1909–1941, 10.1200/JCO.21.00449. 33900808

[10] E. Hitre , B. Budai , Z. Takácsi‐Nagy , et al., “Cetuximab and Platinum‐Based Chemoradio‐ Or Chemotherapy of Patients With Epidermal Growth Factor Receptor Expressing Adenoid Cystic Carcinoma: A Phase II Trial,” British Journal of Cancer 109 (2013): 1117–1122, 10.1038/bjc.2013.468. 23942070 PMC3778310

[11] Y. K. Chae , S. Y. Chung , A. A. Davis , et al., “Adenoid Cystic Carcinoma: Current Therapy and Potential Therapeutic Advances Based on Genomic Profiling,” Oncotarget 6 (2015): 37117–37134, 10.18632/oncotarget.5076. 26359351 PMC4741919

[12] L. E. Miller , V. Au , T. E. Mokhtari , D. Goss , D. L. Faden , and M. A. Varvares , “A Contemporary Review of Molecular Therapeutic Targets for Adenoid Cystic Carcinoma,” Cancers 14 (2022): 992, 10.3390/cancers14040992. 35205740 PMC8869877

[13] Y. Meng , J. Jin , C. Gong , et al., “Phase II Study of Chidamide in Combination With Cisplatin in Patients With Metastatic Triple‐Negative Breast Cancer,” Annals of Palliative Medicine 10 (2021): 11255–11264, 10.21037/apm-21-1139. 34670391

[14] P. H. Goncalves , L. K. Heilbrun , M. T. Barrett , et al., “A Phase 2 Study of Vorinostat in Locally Advanced, Recurrent, or Metastatic Adenoid Cystic Carcinoma,” Oncotarget 8 (2017): 32918–32929, 10.18632/oncotarget.16464. 28415633 PMC5464838

[15] O. Kooshkaki , A. Derakhshani , N. Hosseinkhani , et al., “Combination of Ipilimumab and Nivolumab in Cancers: From Clinical Practice to Ongoing Clinical Trials,” International Journal of Molecular Sciences 21 (2020): 4427, 10.3390/ijms21124427. 32580338 PMC7352976

[16] J. Fayette , C. Even , L. Digue , et al., “NISCAHN: A Phase II, Multicenter Nonrandomized Trial Aiming at Evaluating Nivolumab (N) in Two Cohorts of Patients (Pts) With Recurrent/Metastatic (R/M) Salivary Gland Carcinoma of the Head and Neck (SGCHN), on Behalf of the Unicancer Head & Neck Group,” Journal of Clinical Oncology 37 (2019): 6083, 10.1200/JCO.2019.37.15_suppl.6083.

[17] V. Tchekmedyian , E. J. Sherman , L. Dunn , et al., “A Phase II Trial Cohort of Nivolumab Plus Ipilimumab in Patients (Pts) With Recurrent/Metastatic Adenoid Cystic Carcinoma (R/M ACC),” Journal of Clinical Oncology 37 (2019): 6084, 10.1200/JCO.2019.37.15_suppl.6084. PMC6599407 30939095

[18] Y. K. Chae , M. Othus , S. P. Patel , et al., “Abstract 3418: A Phase II Basket Trial of Dual Anti‐CTLA‐4 and Anti‐PD‐1 Blockade in Rare Tumors (DART) SWOG S1609: The Salivary Gland Tumor Cohort,” Cancer Research 80 (2020): 3418, 10.1158/1538-7445.AM2020-3418.

[19] S. Park , C.‐Y. Ock , H. Kim , et al., “Artificial Intelligence–Powered Spatial Analysis of Tumor‐Infiltrating Lymphocytes as Complementary Biomarker for Immune Checkpoint Inhibition in Non–Small‐Cell Lung Cancer,” Journal of Clinical Oncology 40 (2022): 1916–1928, 10.1200/JCO.21.02010. 35271299 PMC9177249

[20] H. A. Jung , K.‐U. Park , S. Cho , et al., “A Phase II Study of Nivolumab Plus Gemcitabine in Patients With Recurrent or Metastatic Nasopharyngeal Carcinoma (KCSG HN17–11),” Clinical Cancer Research 28 (2022): 4240–4247, 10.1158/1078-0432.CCR-22-1238. 35819451

[21] J. L. Vos , B. Burman , S. Jain , et al., “Nivolumab Plus Ipilimumab in Advanced Salivary Gland Cancer: A Phase 2 Trial,” Nature Medicine 29 (2023): 3077–3089, 10.1038/s41591-023-02518-x. PMC11293616 37620627

[22] Y. Nakamura , M. Nakayama , B. Nishimura , et al., “Case Report: Complete Response of Recurrent and Metastatic Cystadenocarcinoma of the Parotid Gland With a Single Course of Combined Nivolumab and Ipilimumab Therapy,” Frontiers in Oncology 11 (2021): 618201, 10.3389/fonc.2021.618201. 33718174 PMC7952980

[23] R. Ferrarotto , Y. Mitani , D. J. McGrail , et al., “Proteogenomic Analysis of Salivary Adenoid Cystic Carcinomas Defines Molecular Subtypes and Identifies Therapeutic Targets,” Clinical Cancer Research 27 (2023): 852–864, 10.1158/1078-0432.CCR-20-1192. PMC7854509 33172898

[24] D. Liu , J. Benzaquen , L. G. T. Morris , M. Ilié , and P. Hofman , “Mutations in KMT2C, BCOR and KDM5C Predict Response to Immune Checkpoint Blockade Therapy in Non‐Small Cell Lung Cancer,” Cancers 14 (2022): 2816, 10.3390/cancers14112816. 35681795 PMC9179442

[25] U. Mahmood , A. Bang , Y.‐H. Chen , et al., “A Randomized Phase 2 Study of Pembrolizumab With or Without Radiation in Patients With Recurrent or Metastatic Adenoid Cystic Carcinoma,” International Journal of Radiation Oncology 109 (2021): 134–144, 10.1016/j.ijrobp.2020.08.018. PMC9361179 32781104

[26] M. D. Hellmann , L. Paz‐Ares , R. Bernabe Caro , et al., “Nivolumab Plus Ipilimumab in Advanced Non–Small‐Cell Lung Cancer,” New England Journal of Medicine 381 (2019): 2020–2031, 10.1056/NEJMoa1910231. 31562796

[27] M. L. Johnson , B. C. Cho , A. Luft , et al., “Durvalumab With or Without Tremelimumab in Combination With Chemotherapy as First‐Line Therapy for Metastatic Non–Small‐Cell Lung Cancer: The Phase III POSEIDON Study,” Journal of Clinical Oncology 41 (2023): 1213–1227, 10.1200/JCO.22.00975. 36327426 PMC9937097

[28] R. Ferrarotto , L. G. Sousa , L. Feng , et al., “Phase II Clinical Trial of Axitinib and Avelumab in Patients With Recurrent/Metastatic Adenoid Cystic Carcinoma,” Journal of Clinical Oncology 41 (2023): 2843–2851, 10.1200/JCO.22.02221. 36898078 PMC10414730

[29] M. Mohamadpour , E. J. Sherman , A. Kriplani , et al., “A Phase II Study of Lenvatinib Plus Pembrolizumab in Patients With Progressive, Recurrent/Metastatic Adenoid Cystic Carcinoma,” Journal of Clinical Oncology 41 (2023): 6048, 10.1200/JCO.2023.41.16_suppl.6048. PMC6599407 30939095

[30] K. Niwa , D. Kawakita , T. Nagao , et al., “Multicentre, Retrospective Study of the Efficacy and Safety of Nivolumab for Recurrent and Metastatic Salivary Gland Carcinoma,” Scientific Reports 10 (2020): 16988, 10.1038/s41598-020-73965-6. 33046752 PMC7552420

[31] Y. Nagatani , N. Kiyota , T. Yamazaki , et al., “A Phase II Trial of Nivolumab for Patients With Platinum‐Refractory Recurrent or Metastatic Salivary Gland Cancer,” Journal of Clinical Oncology 41 (2023): 6092, 10.1200/JCO.2023.41.16_suppl.6092. PMC13017010 41342548

[32] R. B. Cohen , J.‐P. Delord , T. Doi , et al., “Pembrolizumab for the Treatment of Advanced Salivary Gland Carcinoma: Findings of the Phase 1b KEYNOTE‐028 Study,” American Journal of Clinical Oncology 41 (2018): 1083–1088, 10.1097/COC.0000000000000429. 29462123 PMC6211783

[33] C. Even , J.‐P. Delord , K. A. Price , et al., “Evaluation of Pembrolizumab Monotherapy in Patients With Previously Treated Advanced Salivary Gland Carcinoma in the Phase 2 KEYNOTE‐158 Study,” European Journal of Cancer 171 (2022): 259–268, 10.1016/j.ejca.2022.05.007. 35777186

[34] M. Z. Wojtukiewicz , E. Sierko , D. Hempel , S. C. Tucker , and K. V. Honn , “Platelets and Cancer Angiogenesis Nexus,” Cancer Metastasis Reviews 36 (2017): 249–262, 10.1007/s10555-017-9673-1. 28681240 PMC5557865

[35] B. Li , P. Zhou , Y. Liu , et al., “Platelet‐To‐Lymphocyte Ratio in Advanced Cancer: Review and Meta‐Analysis,” Clinica Chimica Acta 483 (2018): 48–56, 10.1016/j.cca.2018.04.023. 29678631

[36] I. Plesca , A. Tunger , L. Müller , et al., “Characteristics of Tumor‐Infiltrating Lymphocytes Prior to and During Immune Checkpoint Inhibitor Therapy,” Frontiers in Immunology 11 (2020): 00364, 10.3389/fimmu.2020.00364. PMC7064638 32194568

[37] B. Burman , E. J. Sherman , L. Dunn , et al., “A Phase II Trial Cohort of Nivolumab Plus Ipilimumab in Patients (Pts) With Recurrent/Metastatic Salivary Gland Cancers (R/M SGCs),” Journal of Clinical Oncology 39 (2021): 6002, 10.1200/JCO.2021.39.15_suppl.6002.

