# Supplementary material for: Phase II Study of Nivolumab and Ipilimumab for Treatment of Metastatic/Recurrent Adenoid Cystic Carcinoma (ACC) of all Anatomic Sites of Origin and Other Malignant Salivary Gland Tumors
Source: Cancer Med. 2025 Apr 1;14(7):e70724. doi: 10.1002/cam4.70724 (PMC11959299; doi:10.1002/cam4.70724)
Supplement: Supplementary file 4 — Data S1. [file CAM4-14-e70724-s004.docx]

For patients with metastatic/recurrent ACC, the optimal two-stage design to test the null hypothesis that P≤0.450 versus the alternative that P≥0.650 has an expected sample size of 24.70 and a probability of early termination of 0.654. If the drug is not actually effective, there is a 0.048 (target 0.05) probability of concluding that it is effective. If the drug is actually effective, there is a 0.196 (target 0.200) probability of concluding that it is not.
